# Neurobiological mechanisms of TENS-induced analgesia

**DOI:** 10.1016/j.neuroimage.2019.03.077

**Published:** 2019-07-15

**Authors:** W.W. Peng, Z.Y. Tang, F.R. Zhang, H. Li, Y.Z. Kong, G.D. Iannetti, L. Hu

**Affiliations:** a College of Psychology and Sociology, Shenzhen University, Shenzhen, China; b CAS Key Laboratory of Mental Health, Institute of Psychology, Beijing, China; c Department of Psychology, University of Chinese Academy of Sciences, Beijing, China; d Research Center of Brain Cognitive Neuroscience, Liaoning Normal University, Dalian, China; e Neuroscience and Behaviour Laboratory, Istituto Italiano di Tecnologia, Rome, Italy; f Department of Neuroscience, Physiology and Pharmacology, University College London, London, UK; g Department of Pain Management, The State Key Clinical Specialty in Pain Medicine, The Second Affiliated Hospital of Guangzhou Medical University, Guangzhou, China

**Keywords:** Transcutaneous electrical nerve stimulation (TENS), Pain, Analgesia, Electroencephalography (EEG), Resting state, Human

## Abstract

Pain inhibition by additional somatosensory input is the rationale for the widespread use of Transcutaneous Electrical Nerve Stimulation (TENS) to relieve pain. Two main types of TENS produce analgesia in animal models: high-frequency (∼50–100 Hz) and low-intensity ‘conventional’ TENS, and low-frequency (∼2–4 Hz) and high-intensity ‘acupuncture-like’ TENS. However, TENS efficacy in human participants is debated, raising the question of whether the analgesic mechanisms identified in animal models are valid in humans. Here, we used a sham-controlled experimental design to clarify the efficacy and the neurobiological effects of ‘conventional’ and ‘acupuncture-like’ TENS in 80 human volunteers. To test the analgesic effect of TENS we recorded the perceptual and brain responses elicited by radiant heat laser pulses that activate selectively Aδ and C cutaneous nociceptors. To test whether TENS has a long-lasting effect on brain state we recorded spontaneous electrocortical oscillations. The analgesic effect of ‘conventional’ TENS was maximal when nociceptive stimuli were delivered homotopically, to the same hand that received the TENS. In contrast, ‘acupuncture-like’ TENS produced a spatially-diffuse analgesic effect, coupled with long-lasting changes both in the state of the primary sensorimotor cortex (S1/M1) and in the functional connectivity between S1/M1 and the medial prefrontal cortex, a core region in the descending pain inhibitory system. These results demonstrate that ‘conventional’ and ‘acupuncture-like’ TENS have different analgesic effects, which are mediated by different neurobiological mechanisms.

## Highlights

• ‘Acupuncture-like’ TENS produces a spatially-diffuse analgesic effect.
• ‘Conventional’ TENS produces a spatially-localized analgesic effect.
• ‘Acupuncture-like’ TENS results in an enhancement of ongoing α oscillation in S1/M1.
• ‘Acupuncture-like’ TENS results in an enhanced connectivity between S1/M1 and mPFC.
• These two types of TENS are mediated by different neurobiological mechanisms.

## 1 Introduction

It is well-known that rubbing the skin over a bruised area inhibits pain. Yet, the physiological mechanisms of touch-induced analgesia remain unclear (Braz et al., 2014; Mendell, 2014). The analgesic effect of tactile stimulation constitutes the rationale for using Transcutaneous Electrical Nerve Stimulation (TENS) (Sluka and Walsh, 2003), the delivery of electrical stimuli that activate peripheral somatosensory afferents, to relieve both acute and chronic pain (Inui et al., 2006; Rakel et al., 2014). Despite being widely offered as a treatment for pain (Rakel et al., 2014), there is no conclusive evidence that TENS is effective in a number of clinical conditions, and a great deal of confusion about the efficacy of TENS reigns (Bergeron-Vezina et al., 2015; Rakel et al., 2014).

The frequency and the intensity of electrical pulses has been suggested to be one of the crucial determinants of the duration and type of analgesic effect provided by TENS (Johnson and Martinson, 2007). TENS is typically delivered using two distinct sets of stimulus parameters: (1) high frequency (∼50–100 Hz) and low intensity “conventional” TENS, evoking a comfortable, nonpainful tingling sensations (Leonard et al., 2010), and (2) low frequency (∼2–4 Hz) and high intensity “acupuncture-like” TENS, evoking tolerable but painful sensations (Han, 2003). Considering this diversity of stimulus parameters, it is evident that despite being both labeled using the same acronym, the two types of TENS have profoundly different effects on the nervous system. Perhaps unsurprisingly, evidence from animal studies suggests that “conventional” and “acupuncture-like” TENS engage different analgesic mechanisms (Radhakrishnan et al., 2003). “Conventional” TENS is usually related to the gate control theory that high-frequency and low-intensity stimulation of large-diameter Aβ afferents results in a *segmental* inhibition of the transmission of nociceptive information at the dorsal horn level (Melzack and Wall, 1965). On the other hand, “acupuncture-like” TENS (DeSantana et al., 2009; Kalra et al., 2001; Sluka and Walsh, 2003) is more related to the diffuse noxious inhibitory control (DNIC) phenomenon (Le Bars et al., 1992): a strong noxious input causes the release of endogenous opioids in the periaqueductal gray (PAG) and rostral ventral medulla (RVM), which in turn results in a *diffuse* descending inhibition of nociception.

Despite the evidence that the two types of TENS have analgesic effects mediated by distinct mechanisms in animal pain models (Radhakrishnan et al., 2003; Radhakrishnan and Sluka, 2003; Sluka and Walsh, 2003), whether TENS produces analgesia in healthy participants (Barlas et al., 2006; Bergeron-Vezina et al., 2015; Liebano et al., 2011) and chronic pain patients (Ezzo et al., 2000; Oosterhof et al., 2008) remains controversial. This question is particularly relevant when considering the inherent differences between species, as well as the fact that animal TENS effects are typically detected in anaesthetized or “spinal” preparations (Blackburn-Munro, 2004; Garrison and Foreman, 1996; Hu et al., 2015a; Mogil, 2009).

In this study we explored the neurophysiological and perceptual effects of sham and active TENS delivered at either high or low-frequency in a population of 80 healthy human volunteers. While recording both spontaneous and stimulus-evoked brain activities, we tested whether TENS affected subjective ratings of pain intensity and unpleasantness elicited by nociceptive stimulation, as well as whether TENS affected the transient electrocotical responses elicited by the same nociceptive simuli. We also explored whether TENS had a long-lasting effect on brain state, indexed using ongoing electrocortical oscillations.

## 2 Materials and methods

### 2.1 Subjects

A total of 80 healthy, pain-free volunteers who never had TENS before (all right-handed; 40 female; mean age 20.5 ± 1.8 years; age range 18–27 years) were recruited through local advertisement. Subjects were medically screened, and excluded if they had peripheral and central nervous system disease, cardiac pacemaker, chronic pain, or if they were under any type of pain medication. After the screening, subjects were told that the aim of the study was “to investigate the neurophysiological and perceptual effects of some electrical stimulation delivered to the skin”. All subjects gave their written informed consent prior to testing and were paid for their participation. Experimental procedures were approved by the local ethics committee.

### 2.2 Sensory stimulation and experimental design

Electrical stimuli for both active and sham TENS were generated by a constant current electrical stimulator (Sanxia technique Inc., China), and delivered through a pair of surface round electrodes (diameter: 16 mm; inter-electrode distance: 3 cm) placed over the radial nerve at the wrist, either on the left or on the right side. Four different sets of stimulus parameters were used in four experimental groups, as follows. Group 1: high-frequency active TENS; Group 2: low-frequency active TENS; Group 3: high-frequency sham TENS; Group 4: low-frequency sham TENS. Each subject was randomly assigned to one of the four groups. Age and sex were matched between groups. High-frequency (100 Hz) and low-frequency (4 Hz) TENS (both active and sham) consisted of a series of succeeding constant-current square-wave pulses (0.2 ms duration for each pulse). The duration of the TENS session was the same in the active and sham TENS. However, in the active TENS the stimulation lasted for 30 min, whereas in the sham TENS the stimulation lasted for 45 s. In the high-frequency active TENS the stimulus intensity was individually adjusted to elicit a strong but non-painful tingling sensation. In the low-frequency active TENS the stimulus intensity was individually adjusted to elicit a tolerable painful sensation (Bergeron-Vezina et al., 2015). In both high-frequency and low-frequency sham TENS the stimulus intensity was individually adjusted to the thresholds for detecting the electrical stimulus. Importantly, given that the TENS sensation habituates after the first occurrences of the stimulus (Pantaleao et al., 2011), in all conditions (active and sham, high-frequency and low-frequency) the stimulus intensity was determined as follows. First, the desired perceptual outcome of each condition (the strong but non-painful tingling sensation in the high-frequency TENS, the tolerable painful sensation in the low-frequency TENS, and so on) was obtained in three consecutive attempts. Second, the stimulus intensity used in the actual experiment was the highest of the three attempts for each condition. Before the TENS, participants were given the following instruction: “You will now receive 30 min of TENS. This can result in sensations that vary greatly among subjects, and range from intense sensations to much weaker sensations, or even no sensation at all”.

Before and after the TENS procedure, we recorded the brain responses elicited by nociceptive-specific radiant-heat stimuli generated by an infrared neodymium yttrium aluminum perovskite laser with a wavelength of 1.34 μm and a pulse duration of 4 ms (Electronic Engineering, Italy). At this wavelength and pulse duration, laser stimuli activate directly nociceptive terminals in the most superficial skin layers in a synchronized fashion (Iannetti et al., 2004). A He − Ne laser pointed to the area to be stimulated. The laser beam was transmitted via an optic fiber and its diameter was set at approximately 7 mm by focusing lenses. Laser pulses were delivered to a squared area (4 × 4 cm^2^) on the dorsum of each hand (i.e., both ipsilateral and contralateral to the TENS side). After each stimulus, the beam target was shifted by at least 1 cm in a random direction within the squared area, to avoid nociceptor fatigue or sensitization. In a preliminary session, the laser energy was individually determined by increasing the stimulus energy in steps of 0.25 J, until a rating of 7 out of 10 was obtained on a numerical rating scale (NRS) ranging from 0 (no pain) to 10 (pain as bad as it could be).

**Fig. 1:**
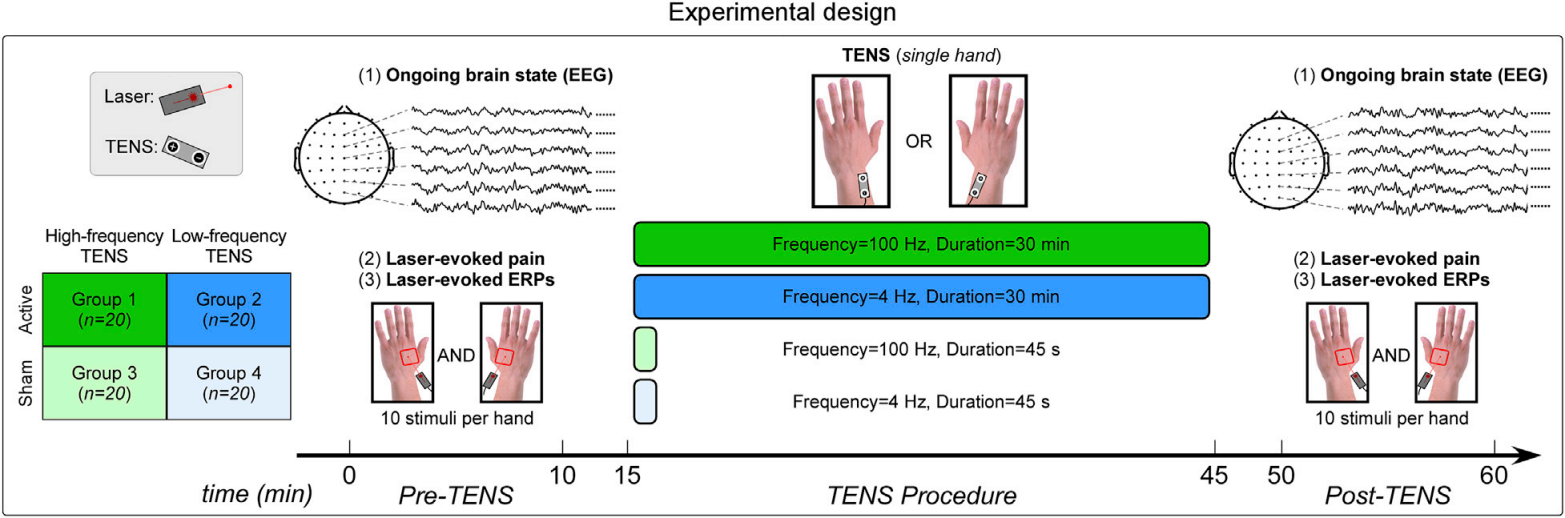
Experimental design. 80 human participants were randomly assigned to four experimental groups (20 subjects per group), as follows. Group 1: high-frequency active TENS; Group 2: low-frequency active TENS; Group 3: high-frequency sham TENS; Group 4: low-frequency sham TENS. High-frequency (100 Hz) and low-frequency (4 Hz) TENS consisted of constant-current square-wave pulses (duration 200 μs) delivered transcutaneously to the radial nerve at the wrist, either on the left or on the right side. Active and sham TENS lasted for 30 min and 45 s respectively. Five minutes before (“Pre-TENS”) and after (“Post-TENS”) TENS, ongoing brain activity was measured using 64-channel EEG. In addition, 20 nociceptive laser stimuli were delivered to participants' hand dorsum, on both sides (10 stimuli per side). After each stimulus, subjects were instructed to rate the intensity and unpleasantness of the perceived pain using a 0–10 numerical rating scale.

A placebo-controlled experimental design was used (Fig. 1). Subjects were randomly assigned to one of the four experimental groups (20 subjects per group). The experiment consisted of three sessions, separated by a 5-min break: Pre-TENS (∼10 min), TENS (∼30 min), and Post-TENS (∼10 min). In both Pre-TENS and Post-TENS sessions, 20 nociceptive laser stimuli of identical energy were delivered to the dorsum of both hands (10 stimuli per hand). The inter-stimulus interval varied randomly between 18 and 20 s. The order of stimulated hand was pseudorandomized, with the constraint that no more than two stimuli could be delivered to the same hand. In half of the subjects of each group the first stimulus was delivered on the left hand; in the other half the first stimulus was delivered on the right hand. Approximately 3 s after each laser stimulus, subjects were asked to verbally report both pain intensity (with the same NRS used in the preliminary energy determination) and pain unpleasantness (using an NRS ranging from 0 [not unpleasant] to 10 [maximally unpleasant]).

It is important to mention that active and sham TENS induced different sensations, and thereforea within-subject design would not have been blinded with respect to the experimental conditions. Thus, we decided to adopt instead a between-subject, placebo-controlled experimental design, in which subjects were randomly assigned to one of four experimental groups. In addition, data from different subjects were collected at different time periods, and subjects were not able to communicate with each other about the sensation felt during the experiment. Still, the fact that active and sham TENS produced different sensations does not allow us to completely rule out the possible influence of unspecific stimulation effects on the observed results.

### 2.3 EEG data collection

Subjects seated in a comfortable chair in a silent room which temperature was maintained between 24 and 26 °C. They were instructed to focus on the stimuli, keep their eyes open, and gaze at a fixation point on the screen. A curtain was used to block the subjects’ view of their forearms. EEG data were collected using 64 Ag—AgCl scalp electrodes placed according to the International 10–20 system (Brain Products GmbH; pass band: 0.01–100 Hz; sampling rate: 1000 Hz). The nose was used as reference, and electrode impedances were kept lower than 10 kΩ. Electrooculographic signals were simultaneously recorded using two surface electrodes, one placed ∼10 mm below the left eye and the other placed ∼10 mm from the outer canthus of the left eye.

### 2.4 EEG data preprocessing

EEG data were preprocessed using EEGLAB (Delorme and Makeig, 2004), an open source toolbox running in the MATLAB environment. Continuous EEG data were band-pass filtered between 1 and 30 Hz. EEG epochs were extracted using a window analysis time of 1500 ms (500 ms pre-stimulus and 1000 ms post-stimulus), and baseline corrected using the pre-stimulus interval. Trials contaminated by eye-blinks and movements were corrected using an Independent Component Analysis algorithm (Delorme and Makeig, 2004). To compare the responses elicited by laser stimulation delivered contralaterally and ipsilaterally to the TENS side, the EEG data from participants receiving TENS on their right hand were flipped along the medio-lateral axis (Peng et al., 2017).

### 2.5 Laser-evoked brain potentials (LEPs)

For each subject, session, and stimulated hand, single-trial LEP waveforms in the time domain were averaged together. This procedure yielded four average waveforms for each subject, time-locked to the onset of laser stimulation. Peak latencies and amplitudes of N2 and P2 waves, defined as the most negative and positive deflections between 150 and 500 ms after stimulus onset respectively, were measured from each single-subject average waveform, at Cz. Peak latency and amplitude of N1 wave, defined as the most negative deflection preceding the N2 wave, were measured at the central electrode contralateral to the stimulated side (Cc), referenced to Fz (Valentini et al., 2012; Hu et al., 2010). Single-subject average LEP waveforms were subsequently averaged across subjects composing each of the four experimental groups, to obtain group-level LEP waveforms. Group-level scalp topographies at the peak latency of N1, N2, and P2 waves were computed by spline interpolation.

We used a time-frequency analysis to explore both phase-locked and non-phase-locked brain responses elicited by laser stimuli. Time-frequency distributions (TFDs) of EEG trials were estimated using a windowed Fourier transform (WFT) with a fixed 250-ms Hanning window. WFT yielded, for each trial, a complex time-frequency estimate F(t,f) at each time-frequency point (t,f), extending from −500 ms to 1000 ms (in steps of 1 ms) in the time domain, and from 1 to 30 Hz (in steps of 1 Hz) in the frequency domain. The resulting spectrogram, P(t,f) = |F(t,f)|^2^, represents the signal magnitude as a joint function of time and frequency at each time-frequency point. The spectrogram was baseline-corrected (reference interval: from −400 to −100 ms) at each frequency using the subtraction approach (Hu et al., 2014). As described in several previous studies (Mouraux et al., 2003; Schulz et al., 2011), TFDs elicited by laser stimuli contain both phase-locked (event-related potential, ERP) and non-phase-locked (event-related desynchronization at alpha frequencies, α-ERD) responses. To extract the magnitude of time-frequency brain responses, we used region-of-interests (ROIs) defined on the basis of previous observations (Hu et al., 2015b; Iannetti et al., 2008): ERP (100–500 ms, 1–10 Hz) and α-ERD (500–1000 ms, 8–12 Hz). Magnitudes of each time-frequency feature were calculated by computing the mean of the top 20% time-frequency points displaying the highest increase (for ERP) or decrease (for α-ERD) for each subject in each experimental condition (Mouraux and Iannetti, 2008).

### 2.6 Ongoing EEG oscillations

#### 2.6.1 Scalp-level analysis

Prestimulus EEG signals were extracted from a time window ranging from −4000 ms to 0 ms relative to laser stimulus onset. For each subject and session, prestimulus EEG signals were transformed to the frequency domain using a discrete Fourier transform, yielding an EEG spectrum ranging from 1 to 30 Hz. Single-subject EEG spectra were averaged across subjects composing each of the four experimental groups, to obtain group-level prestimulus EEG spectra. Since prestimulus alpha oscillations have been showed to influence both perception and brain responses elicited by subsequent sensory stimuli (Babiloni et al., 2006; Tu et al., 2016), we tested the *a priori* hypothesis that possible pain modulation caused by TENS was mediated by the effect of prestimulus alpha oscillations on subsequent laser-evoked pain ratings and EEG responses.

#### 2.6.2 Source-level analysis

To localize the sources of TENS-induced changes of brain oscillations we used a beamforming algorithm known as dynamic imaging of coherent sources (Gross et al., 2001), implemented in the open-source Matlab toolbox FieldTrip (Oostenveld et al., 2011). This algorithm computes a spatial filter based on a leadfield matrix (i.e., the matrix of coefficients that maps current sources to potential differences at the scalp) and a cross-spectral density matrix, where (1) the leadfield matrix was computed for a three-dimensional grid with a 1-cm resolution, using a realistically shaped three-shell boundary-element volume conduction model based on the Montreal Neurological Institute template brain, and (2) the cross-spectral density matrix was computed for each of four frequency bands (i.e., delta: 1–4 Hz, theta: 4–8 Hz, alpha: 8–12 Hz, beta: 12–30 Hz) using a multitaper frequency transformation. Time courses of oscillation power for each grid point in source space were computed through multiplying scalp-level time-frequency data by the spatial filter. The estimated power in source space yielded an estimate of the neural sources responsible for each oscillation band, for each session and experimental group. TENS-induced changes of power for each grid point were evaluated as the percentage changde of alpha power in the post-TENS session relative to the pre-TENS session.

To test statistically whether TENS had an effect on ongoing brain oscillations at bilateral primary sensorimotor cortices (S1/M1), we performed the following ROI-based analysis. Source-level ROIs were first defined using the Automated Anatomical Labeling (AAL) brain template (Tzourio-Mazoyer et al., 2002). Specifically, the S1/M1 ipsilateral to TENS was identified using the regions labeled as “Precentral_L” and “Postcentral_L” in the AAL template. The S1/M1 contralateral to TENS was identified using the regions labeled as “Precentral_R” and “Postcentral_R” in the AAL template. For each subject and ROI, the changes of alpha oscillations were obtained by calculating the percentage change of mean alpha power across all voxels of the ROI in the post-TENS session relative to the pre-TENS session. Statistical comparisons were performed on these percentage changes of alpha oscillations, separately for each ROI.

#### 2.6.3 Functional connectivity analysis

To assess the effect of TENS-induced changes of ongoing brain state on the descending pain inhibitory system, we characterized the functional connectivity between bilateral sensorimotor cortices (S1/M1, i.e., the brain areas where TENS induced changes of alpha oscillations) and the medial prefrontal cortex (mPFC, which is a core region in the descending pain inhibitory system, anatomically connected with the brainstem periaqueductal gray, PAG (Kucyi and Davis, 2015)) within the alpha frequency range. The S1/M1 ROIs were identified as described in the previous paragraph. The mPFC ROI was identified by the regions labeled as “Frontal_Med_Orb_L”, “Frontal_Med_Orb_R”, “Frontal_Sup_Medial_L”, and “Frontal_Sup_Medial_R” in the AAL template (Tzourio-Mazoyer et al., 2002). For each of these ROIs, we calculated the time courses of prestimulus alpha oscillations from −4000 ms to 0 ms relative to laser stimulus onset, by averaging the source-level data across all voxels within the ROI. Their functional connectivity was quantified using the estimation of the linear time-invariant relationship between time series (i.e., their coherence (Gross et al., 2001)). Specifically, the coherence was computed as the squared cross-spectrum of two time series, divided by the power spectra of both time series (Gross et al., 2001). This analysis yields a value between 0 (indicating no linear relationship) and 1 (indicating perfect linear relationship). Moreover, we used the directed transfer function (DTF) method (Kaminski and Blinowska, 1991) to investigate the relationship between bilateral S1/M1 and mPFC. This allowed us to verify the coherence results and to test more comprehensively the research hypothesis. DTF is derived from the Granger causality concept (Granger, 1969; Kaminski et al., 2001) and has been demonstrated to quantify effectively the strength of directed functional connectivity between brain regions (Astolfi et al., 2005; Babiloni et al., 2005; Lu et al., 2012; Xu et al., 2015). TENS-induced changes of functional connectivity were estimated by subtracting both the coherence and DTF measures of the Pre-TENS session from those of the Post-TENS session.

### 2.7 Statistical analysis

The possible effect of TENS on both perceptual and electrophysiological responses elicited by nociceptive stimulation was evaluated by calculating the difference of each measure between sessions (Post-TENS *minus* Pre-TENS). The resulting differences were compared using a three-way analysis of variance (ANOVA), with two between-subjects factors (“TENS frequency”: high-frequency and low-frequency; “condition”: active and sham TENS) and one within-subject factor (“side”: laser stimulation of the hand dorsum ipsilateral and contralateral to the TENS side). When there was a significant three-way interaction, we performed a post hoc two-way ANOVA separately for high-frequency and low-frequency TENS. When there was a significant two-way interaction between “condition” and “side”, we performed a further post hoc paired-sample t-tests to compare the changes elicited by laser stimuli delivered to hand ipsilateral and contralateral to the TENS side, separately for active and sham TENS conditions.

To test whether TENS had an effect on the ongoing brain state, we performed a two-way ANOVA with two between-subject factors (“TENS frequency”: high-frequency and low-frequency; “condition”: active and sham TENS) on (1) the amplitude of the ongoing alpha oscillations measured both at scalp and source levels, and (2) the functional connectivity (estimated using both coherence and DTF measures) between bilateral S1/M1 and mPFC. When the interaction between the two factors was significant, we performed post hoc independent-sample t-tests to compare the active TENS with the sham TENS condition, separately for high-frequency and low-frequency TENS.

## 3 Results

### 3.1 Effect of TENS on subjective pain reports

**Fig. 2:**
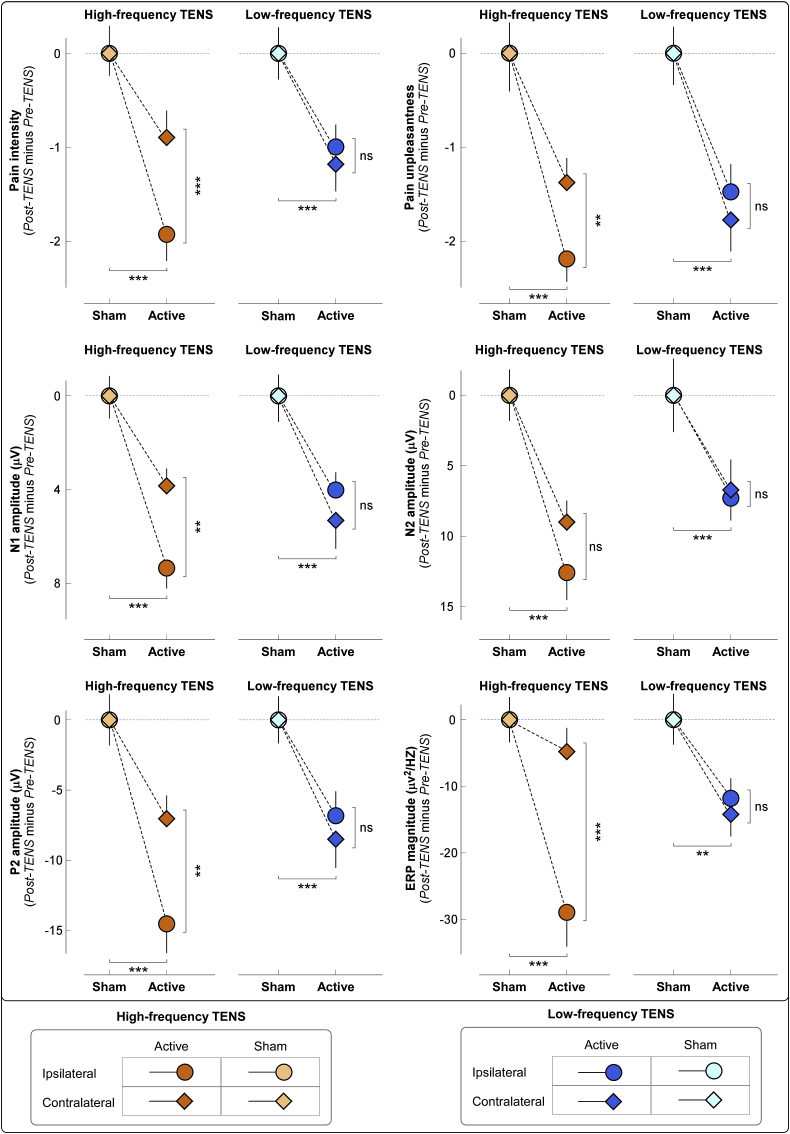
Effects of different TENS type on laser-elicited pain perception and brain responses. Effects of TENS on laser-evoked pain intensity and unpleasantness (*top plots*) and brain responses (*middle and bottom plots*) are evaluated as difference between Pre-TENS and Post-TENS sessions (Post-TENS *minus* Pre-TENS, normalized by subtracting the respective sham data for displaying purpose; statistical results from non-normalized data are reported in the main text). For both TENS types, the decrease of laser-elicited pain perception and brain responses was significantly larger in the active condition than that in the sham condition. High-frequency TENS induced a larger decrease of both pain perception and brain responses when laser stimuli were delivered to the hand ipsilateral to the TENS side than that contralateral to the TENS side (*: p ​< ​0.05; **: p ​< ​0.01; ***: p ​< ​0.001). In contrast, the decrease in pain perception and brain responses induced by low-frequency TENS was similar when laser stimuli were delivered to the hand ipsilateral and contralateral to the TENS side (ns: not significant). Data are mean ​± ​SEM.

TENS induced consistent changes in ratings of both intensity and unpleasantness of the pain elicited by nociceptive laser stimulation (Fig. 2, top panel). Results of the three-way ANOVA are summarized in Table 1. For both pain intensity and unpleasantness, three-way ANOVAs showed strong evidence for a main effect of “condition” (intensity: F_*(1,76)*_ = 27.37, p < 0.001, ɳ^2^_p_ = 0.27; unpleasantness: F_*(1,76)*_ = 35.87, p < 0.001, ɳ^2^_p_ = 0.32) and moderate evidence for a main effect of “side” (intensity: F_*(1,76)*_ = 10.41, p = 0.002, ɳ^2^_p_ = 0.12; unpleasantness: F_*(1,76)*_ = 10.71, p = 0.002, ɳ^2^_p_ = 0.12). These two main effects indicate that both pain intensity and unpleasantness were reduced in the active vs sham TENS, as well as when pain was elicited by laser stimuli delivered ipsilaterally vs contralaterally to the TENS side. There was also weak evidence for a significant three-way interaction (intensity: F_*(1,76)*_ = 5.90, p = 0.02, ɳ^2^_p_ = 0.07; unpleasantness: F_*(1,76)*_ = 4.32, p = 0.04, ɳ^2^_p_ = 0.05).

**Table 1:** Three-way ANOVA with two between-subject factors (“TENS frequency”: high-frequency and low-frequency; “condition”: active and sham TENS) and one within-subject factor (“side”: laser stimuli delivered to hand dorsum ipsilateral and contralateral to TENS side) to assess TENS induced changes (Pre-TENS *minus* Post-TENS) of laser-elicited pain perception (pain intensity and unpleasantness) and brain responses (time domain: N1, N2, and P2 latencies and amplitudes; time-frequency domain: ERP and α-ERD magnitudes).

Three-way ANOVA		Pain intensity	Pain unpleasantness	N1 latency	N1 amplitude	N2 latency	N2 amplitude	P2 latency	P2 amplitude	ERP magnitude	α-ERD magnitude
TENS frequency		F = 0.54	F = 1.40	F = 2.75	F = 0.004	**F** = 4.51^*^	F = 0.16	F = 1.63	F = 0.06	F = 1.58	F = 0.19
Condition		**F** = 27.37^***^	**F** = 35.87^***^	**F** = 8.05^***^	**F** = 42.79^***^	F = 2.80	**F** = 29.56^***^	**F** = 8.03^**^	**F** = 40.94^***^	**F** = 33.02^***^	F = 0.65
Side		**F** = 10.41^***^	**F** = 10.71^***^	F = 0.61	**F** = 8.16^**^	F = 2.34	**F** = 4.33^*^	**F** = 15.07***	F = 3.41	F = 1.96	F = 0.99
TENS frequency × condition		F = 0.46	F = 0.08	F = 0.04	F = 0.35	F = 0.004	F = 1.33	F = 1.58	F = 1.17	F = 0.55	F = 0.40
TENS frequency × side		F = 0.11	F = 0.78	F = 1.09	F = 0.06	F = 1.91	F = 2.84	**F** = 5.22*	**F** = 4.28^*^	F = 1.87	F = 1.03
Condition × side		F = 2.86	F = 0.92	F = 0.72	F = 1.16	F = 2.70	F = 0.78	F = 3.03	F = 1.75	**F** = 4.34^*^	F = 0.17
TENS frequency × condition × side		**F** = 5.90^*^	**F** = 4.32^*^	F = 0.07	**F** = 5.48^*^	F = 1.68	F = 0.41	F = 0.06	**F** = 4.34^*^	**F** = 6.47^**^	F = 0.96
Post hoc two-way ANOVA											
High-frequency TENS	condition	**F** = 15.09^***^	**F** = 18.02^***^	**--**	**F** = 29.93^***^	**--**	**--**	**--**	**F** = 28.90^***^	**F** = 23.85^***^	**--**
	side	**F** = 9.87^**^	**F** = 13.21^***^	**--**	**F** = 5.93^*^	**--**	**--**	**--**	**F** = 6.99^**^	F = 2.93	**--**
	condition × side	**F** = 13.21^***^	**F** = 7.05^**^	**--**	**F** = 7.17^**^	**--**	**--**	**--**	**F** = 5.29^*^	**F** = 8.19^**^	**--**
Low-frequency TENS	condition	**F** = 12.30^***^	**F** = 17.91^***^	**--**	**F** = 15.39^***^	**--**	**--**	**--**	**F** = 13.69^***^	**F** = 11.20^**^	**--**
	side	F = 3.08	F = 2.12	**--**	F = 2.87	**--**	**--**	**--**	F = 0.03	F = 0.001	**--**
	condition × side	F = 0.20	F = 0.47		F = 0.67	**--**	**--**	**--**	F = 0.32	F = 0.15	**--**

*: p ​< ​0.05; **: p ​< ​0.01; ***: p ​< ​0.001.

To interpret the three-way interaction, we performed a post hoc two-way ANOVA using “condition” and “side” as factors, which showed the following results. (1) For high-frequency TENS, there was moderate to strong evidence for a main effect of “condition” (intensity: F_*(1,38)*_ = 15.09, p < 0.001, ɳ^2^_p_ = 0.28; unpleasantness: F_*(1,38)*_ = 18.02, p < 0.001, ɳ^2^_p_ = 0.32) and “side” (intensity: F_*(1,38)*_ = 9.87, p = 0.003, ɳ^2^_p_ = 0.21; unpleasantness: F_*(1,38)*_ = 13.21, p = 0.001, ɳ^2^_p_ = 0.26), as well as moderate evidence for a two-way interaction (intensity: F_*(1,38)*_ = 13.21, p = 0.001, ɳ^2^_p_ = 0.26; unpleasantness: F_*(1,38)*_ = 7.05, p = 0.01, ɳ^2^_p_ = 0.16). Post hoc paired-sample t-tests showed that the active TENS decreased pain more when laser stimuli were delivered to the hand ipsilateral to the TENS side (intensity: p = 0.004; unpleasantness: p = 0.003; Fig. 2, top panel). (2) For low-frequency TENS, there was only a strong main effect of “condition” (intensity: F_*(1,38)*_ = 12.30, p = 0.001, ɳ^2^_p_ = 0.25; unpleasantness: F_*(1,38)*_ = 17.91, p < 0.001, ɳ^2^_p_ = 0.32).

### 3.2 Laser-evoked brain responses

**Fig. 3:**
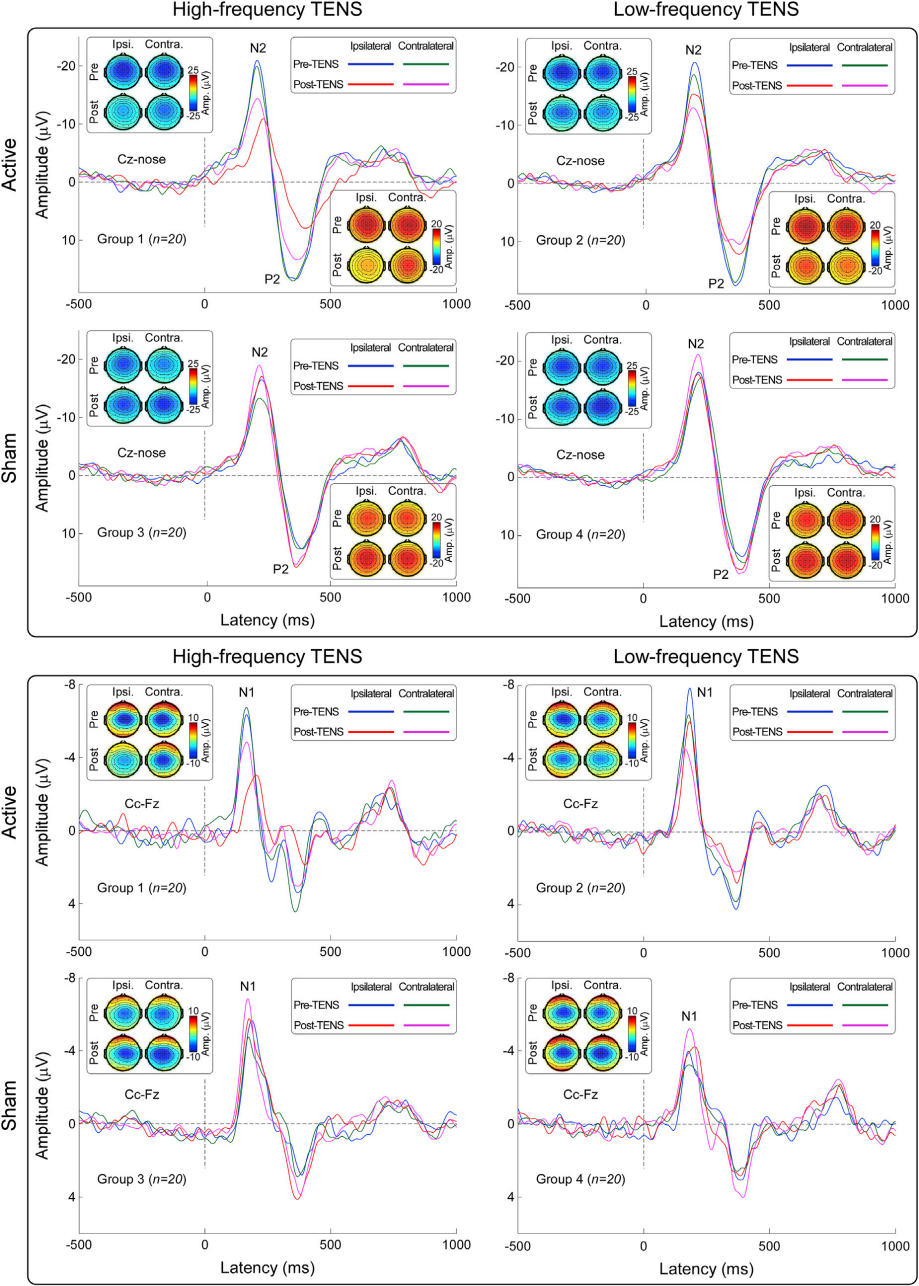
Group-level laser-evoked responses in the time domain. Group-level waveforms and scalp topographies of N2 and P2 waves (Cz-nose, top panel), as well as N1 wave (Cc-Fz, bottom panel), are displayed for each experimental group. In each experimental group, LEPs elicited by stimulation of the hand ipsilateral and contralateral to the TENS side in the Pre-TENS and Post-TENS sessions are superimposed. Scalp topographies are plotted at the peak latency of the N1,N2, and P2 waves.

Group-level LEP waveforms and scalp topographies of N1, N2, and P2 waves in the time domain are shown in Fig. 3. In line with previous studies (Mouraux and Iannetti, 2009; Valentini et al., 2012), scalp topographies of the N1 wave were maximal at central electrodes contralateral to the stimulated hand, scalp topographies of the N2 wave were maximal at the vertex and extended bilaterally towards temporal regions, and scalp topographies of the P2 wave were more centrally distributed.

We observed strong evidence for an effect of TENS on LEP amplitudes (Fig. 2, middle and bottom panels). Results of the three-way ANOVA are summarized in Table 1. For both N1 and N2 amplitudes, there was strong evidence for a main effect of “condition” (N1: F_*(1,76)*_ = 42.79, p < 0.001, ɳ^2^_p_ = 0.36; N2: F_*(1,76)*_ = 29.56, p < 0.001, ɳ^2^_p_ = 0.28) and weak-to-moderate evidence for a main effect of “side” (N1: F_*(1,76)*_ = 8.16, p = 0.006, ɳ^2^_p_ = 0.10; N2: F_*(1,76)*_ = 4.33, p = 0.04, ɳ^2^_p_ = 0.05), indicating that N1 and N2 amplitudes were reduced in the active vs sham TENS (N1: −5.13 μV; N2: −8.90 μV), as well as when they were evoked by laser stimuli delivered ipsilaterally vs contralaterally to the TENS side (N1: −1.47 μV; N2: −2.45 μV). For the P2 amplitude, three-way ANOVA showed a strong main effect of “condition” (F_*(1,76)*_ = 40.94, p < 0.001, ɳ^2^_p_ = 0.35), indicating that P2 amplitudes were significantly reduced in the active vs sham TENS (−9.23 μV). For both N1 and P2 amplitudes, there was weak evidence for a three-way interaction (N1: F_*(1,76)*_ = 5.48, p = 0.02, ɳ^2^_p_ = 0.07; P2: F_*(1,76)*_ = 4.34, p = 0.04, ɳ^2^_p_ = 0.05).

To interpret the three-way interaction of the N1 and P2 amplitudes, we performed a post hoc two-way ANOVA using “condition” and “side”, which showed the following results. (1) For high-frequency TENS, there was strong evidence for main effect of “condition” (N1: F_*(1,38)*_ = 29.93, p < 0.001, ɳ^2^_p_ = 0.44; P2: F_*(1,38)*_ = 28.90, p < 0.001, ɳ^2^_p_ = 0.43) and weak evidence for a main effect of “side” (N1: F_*(1,38)*_ = 5.93, p = 0.02, ɳ^2^_p_ = 0.13; P2: F_*(1,38)*_ = 6.99, p = 0.01, ɳ^2^_p_ = 0.16), as well as a weak two-way interaction (N1: F_*(1,38)*_ = 7.17, p = 0.01, ɳ^2^_p_ = 0.16; P2: F_*(1,38)*_ = 5.29, p = 0.03, ɳ^2^_p_ = 0.12). Post hoc paired-sample t-tests showed that active TENS decreased N1 and P2 amplitudes more when laser stimuli were delivered to the hand ipsilateral to the TENS side (N1: p = 0.006; P2: p = 0.01) (Fig. 2, middle and bottom panels). (2) For low-frequency TENS, there was only strong evidence for a significant main effect of “condition” (N1: F_*(1,38)*_ = 15.39, p < 0.001, ɳ^2^_p_ = 0.29; P2: F_*(1,38)*_ = 13.69, p = 0.001, ɳ^2^_p_ = 0.27).

**Fig. 4:**
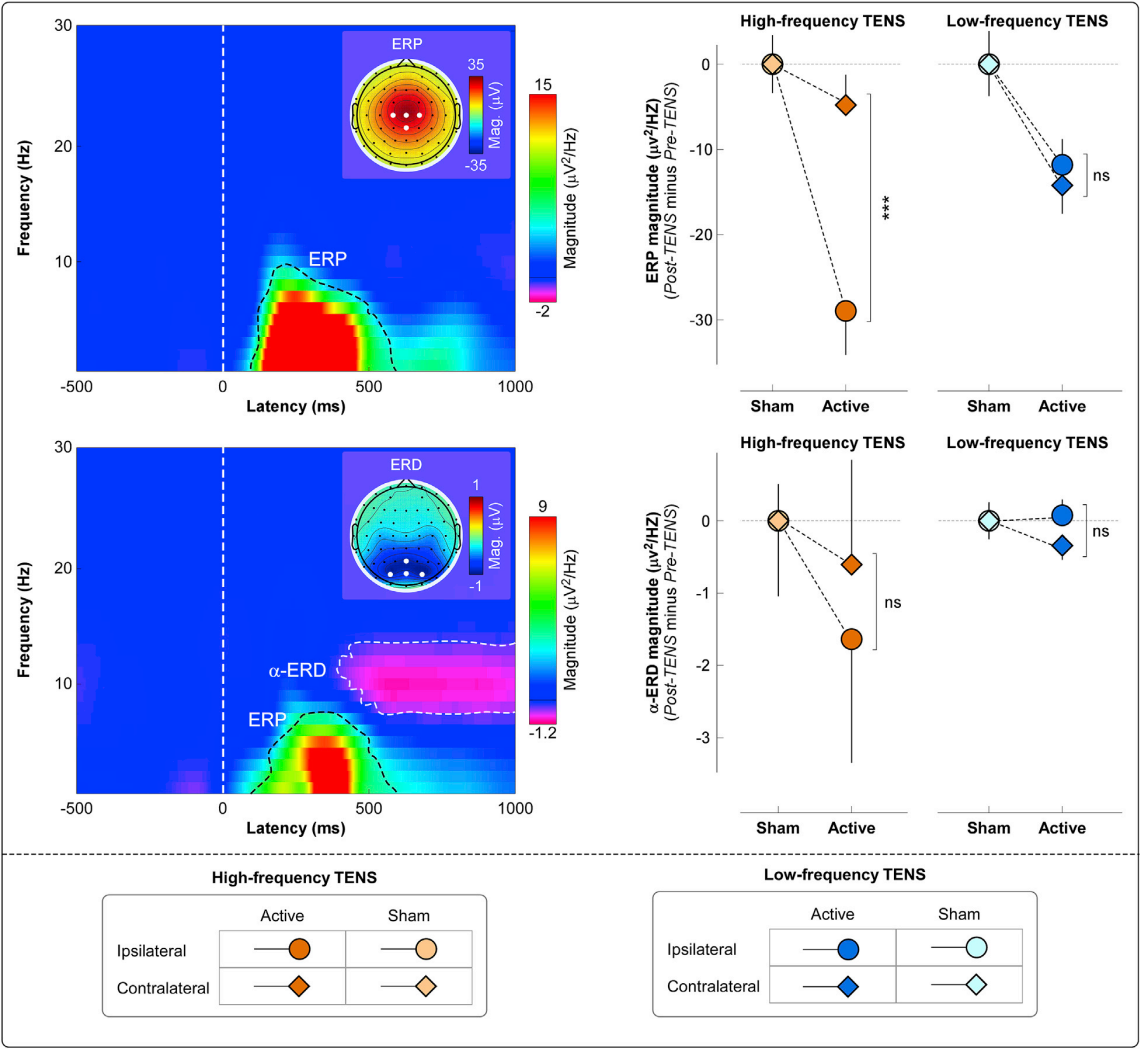
Group-level laser-elicited time-frequency responses. *Left panel:* Group-level time-frequency distributions and scalp topographies of ERP and α-ERD responses, averaged across experimental groups and conditions. The color scale represents the increase or decrease of the oscillatory magnitude, relative to a prestimulus interval (−400 to −100 ms). The displayed time-frequency distributions contain both phase-locked (ERP: 100–500 ms, 1–10 Hz) and non-phase-locked brain responses (α-ERD: 500–1000 ms, 8–12 Hz), highlighted by the dashed lines. ERP and α-ERD magnitudes were measured at central (*top left*) and parietal-occipital (*bottom left*) electrodes respectively. Electrodes showing the maximal response for each time-frequency feature are highlighted in white in the scalp topographies. *Right panel:* The effect of active TENS on the magnitude of the ERP (*top right*) and α-ERD response (*bottom right*) was expressed as difference between Pre-TENS and Post-TENS sessions (Post-TENS *minus* Pre-TENS, normalized by subtracting the respective sham data for displaying purpose; statistical results from non-normalized data are reported in the main text). Data are mean ± SEM.

Group-level time-frequency distributions, together with the scalp topographies of the ‘ERP’ and ‘α-ERD’ responses are shown in Fig. 4. Consistently with previous studies (Mouraux et al., 2003; Schulz et al., 2011), laser stimuli elicited a large phase-locked response (ERP: 100–500 ms, 1–10 Hz, maximal at central midline electrodes) and a clear non-phase-locked response (α-ERD: 500–1000 ms, 8–12 Hz, maximal at parietal-occipital electrodes, bilaterally).

We found strong evidence for an effect of active TENS on ERP magnitude, but not on α-ERD magnitude (Fig. 4). For ERP magnitude, three-way ANOVA showed a strong main effect of “condition” (F_*(1, 76)*_ = 33.02, p < 0.001, ɳ^2^_p_ = 0.30), indicating that ERP was reduced in the active TENS vs sham (−14.94 μV^2^/Hz). There was weak evidence for a two-way interaction between “condition” and “side” (F_*(1, 76)*_ = 4.34, p = 0.04, ɳ^2^_p_ = 0.05), and for a three-way interaction between all factors (F_*(1, 76)*_ = 6.47, p = 0.01, ɳ^2^_p_ = 0.08). In contrast, α-ERD magnitude was not modulated by experimental factors, as indicated by the lack of main effects or their interactions. Results of the three-way ANOVA are summarized in Table 1.

To interpret the three-way interaction on the ERP magnitude, we performed a post hoc two-way ANOVA using “condition” and “side” as factors, which showed the following results. (1) For high-frequency TENS, there was strong evidence for a main effect of “condition” (F_*(1, 38)*_ = 23.85, p < 0.001, ɳ^2^_p_ = 0.39) and moderate evidence for a two-way interaction (F_*(1, 38)*_ = 8.19, p = 0.007, ɳ^2^_p_ = 0.18). Post hoc paired-sample t-tests showed that active TENS decreased ERP magnitude more when laser stimuli were delivered to the hand ipsilateral to the TENS side (p < 0.001; Fig. 2, bottom right panel). (2) For low-frequency TENS, there was only moderate-to-strong evidence for a main effect of “condition” (F_*(1, 38)*_ = 11.20, p = 0.002, ɳ^2^_p_ = 0.23).

In summary, both psychophysical and electrophysiological results showed that the antinociceptive effects of high-frequency TENS were maximal when nociceptive stimuli were given homotopically, i.e., to the same hand where TENS had been delivered. In contrast, low-frequency TENS produced a more spatially diffuse analgesia, also present when nociceptive stimuli were given heterotopically, i.e., to the hand opposite to the TENS side.

### 3.3 Effect of TENS on brain state

**Fig. 5:**
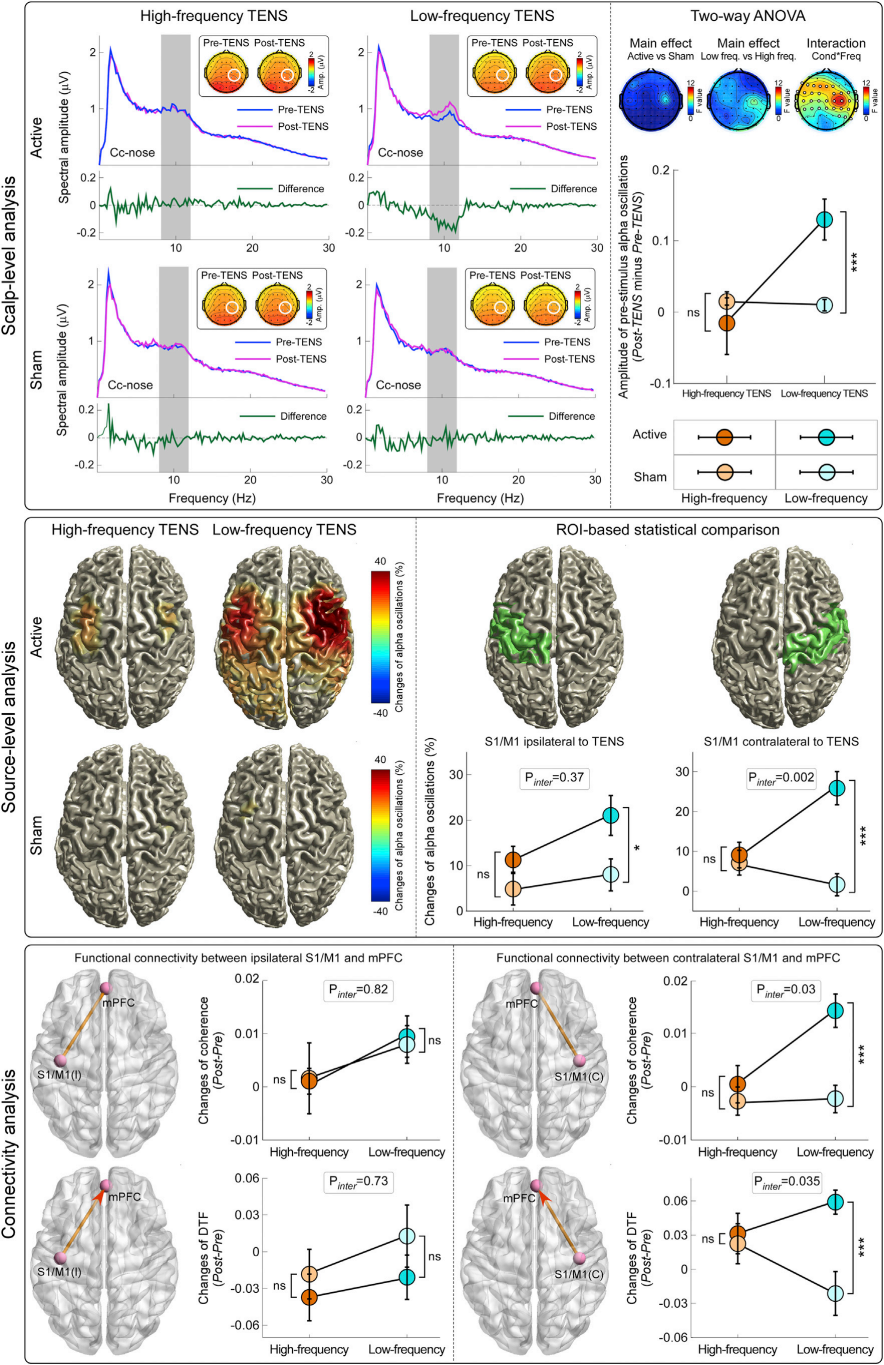
Effects of different TENS type on ongoing brain state.*Top left panel:* Broadband ongoing EEG oscillations in the four experimental groups. Green waveforms show the difference between the Pre-TENS (blue) and Post-TENS (purple) conditions. Only in the low-frequency active TENS group there was a significant difference in the amplitude of alpha oscillations (8–12 Hz, gray area). Scalp maps show the topographical distribution of alpha oscillation amplitude in the four groups. *Top right panel:* Changes of ongoing alpha oscillations (Post-TENS minus Pre-TENS) were compared across-groups using two-way ANOVA with two between-subject factors (‘TENS frequency’ and ‘condition’). There was a significant ‘TENS frequency’ ​× ​‘condition’ interaction at bilateral central electrodes (electrodes with FDR-corrected p ​< ​0.05 are shown in white), maximal on the electrodes overlying the primary sensorimotor cortex (S1/M1) contralateral to the TENS side. Ongoing alpha oscillations were increased in the post-TENS period only in the low-frequency active TENS group (ns: not significant; ***: p ​< ​0.001). Data are mean ​± ​SEM. *Middle panel:* Source-level percentage changes of alpha oscillations in the four experimental groups. Only low-frequency active TENS significantly enhanced alpha power in the bilateral S1/M1. Two-way ANOVA revealed a significant ‘TENS frequency’ ​× ​‘condition’ interaction in the S1/M1 contralateral to the TENS side (p ​= ​0.002, middle right; ns: not significant; *: p<0.05; ***: p ​< ​0.001). Note that the source-level plots displayed in the left part are descriptive: they show the voxels whose absolute percentage change of alpha oscillations (alpha power in the post-TENS session relative to the pre-TENS session) was >10%. *Bottom panel:* Changes of functional connectivity between S1/M1 and mPFC, in the four experimental groups (Post-TENS minus Pre-TENS). Two-way ANOVA revealed that low-frequency TENS caused a significant enhancement of functional connectivity (indexed by both coherence and DTF measures) between the *contralateral* S1/M1 and mPFC (ns: not significant; ***: p ​< ​0.001).

When ongoing brain activity was measured at scalp level, two-way ANOVA showed strong evidence for an interaction between the factors “condition” and “TENS frequency” (Fig. 5, top right panel). A false discovery rate procedure was used to correct the significance level (p value) to account for multiple comparisons across electrodes (Benjamini and Hochberg, 1995). This interaction was maximal at the central electrodes contralateral to the TENS side (C2, C4, CP2, and CP4). Fig. 5 (top left panel) shows the group-level spectral power of the prestimulus EEG measured at these electrodes, in the Pre-TENS and Post-TENS sessions of each group. While scalp topographies of alpha oscillations were, as expected, maximal at parietal-occipital regions for all sessions and groups (Capotosto et al., 2017), there was a strong “condition” × “TENS frequency” interaction at bilateral central regions, showing a maximum over central electrodes *contralateral* to TENS side (Fig. 5, top right panel; F_*(1,38)*_ = 10.88, p = 0.001, ɳ^2^_p_ = 0.13). To interpret this two-way interaction we performed post hoc independent-sample t-tests, which showed that the increase of alpha amplitude (Post-TENS *minus* Pre-TENS) was larger in the active TENS condition than in the sham condition for the low-frequency TENS (p = 0.001), but not for the high-frequency TENS (p = 0.87).

When ongoing brain activity was estimated in source space (Fig. 5, middle panel), two-way ANOVA of the change of alpha oscillations at *contralateral* S1/M1 revealed moderate-to-strong evidence for an interaction between the factors “condition” and “TENS frequency” (F_*(1,38)*_ = 10.05, p = 0.002, ɳ^2^_p_ = 0.12), indicating that the alpha enhancement was greater in the active TENS than in the sham condition of the low-frequency TENS (p < 0.001), but not of the high-frequency TENS (p = 0.68). In contrast, two-way ANOVA of the change of alpha oscillations at *ipsilateral* S1/M1 revealed moderate evidence for a main effect of “condition” (F_*(1,38)*_ = 6.78, p = 0.01, ɳ^2^_p_ = 0.08), indicating that active TENS induced greater enhancement of alpha power than sham TENS.

The analysis of functional connectivity demonstrated that TENS-induced changes in alpha oscillations in S1/M1 affected the ongoing activity of mPFC, a key area of the descending pain inhibition system (Fig. 5, bottom panel). Two-way ANOVA showed weak evidence for a significant interaction between factors “condition” and “TENS frequency” (F_*(1,38)*_ = 4.65, p = 0.03, ɳ^2^_p_ = 0.06), suggesting that the increase of functional connectivity indexed by coherence between contralateral S1/M1 and mPFC was larger in the active TENS condition than in the sham condition of the low-frequency TENS (p < 0.001), but not of the high-frequency TENS (p = 0.49).

When assessing the information flow from the contralateral S1/M1 to the mPFC, we observed weak evidence for a significant interaction between factors “condition” and “TENS frequency” (F_(1,38)_ = 4.60, p = 0.035, ɳ^2^_p_ = 0.057), suggesting that the TENS-induced increase of information flow (indexed by the DTF measure) was larger in the active TENS condition than in the sham condition for low-frequency TENS (p < 0.001), but not for high-frequency TENS (p = 0.72). In contrast, no significant main effect or interaction was observed when assessing the information flow from ipsilateral S1/M1 to mPFC, as well as from mPFC to either contralateral or ipsilateral S1/M1.

These results show that low-frequency TENS induced a clear change in the ongoing brain state, namely a sustained increase of the magnitude of alpha oscillations in the primary sensorimotor cortex contralateral to the hand where the low-frequency TENS was delivered. This TENS-induced change of state of the contralateral S1/M1 cortex resulted in an increased functional connectivity between S1/M1 and mPFC.

## 4 Discussion

In the present study, we used a sham-controlled design to investigate the neurobiological and analgesic effects of the two most common types of TENS used in animal models and human clinical studies (Fig. 1).

We obtained three main results. First, both ‘conventional’ (low-frequency and high-intensity) and ‘acupuncture-like’ (high-frequency and low-intensity) TENS produced an analgesic effect stronger than in the sham condition. However, the analgesic effect of high-frequency and low-intensity TENS was maximal when nociceptive stimuli were delivered *homotopically*, i.e., to the same hand that received the TENS. In contrast, low-frequency and high-intensity TENS produced a spatially diffuse analgesic effect, equally strong regardless of whether nociceptive laser stimuli were delivered to the hand ipsilateral or contralateral to the TENS side (Fig. 2). Second, the recording of transient laser-evoked brain responses provided a physiological support to the modulation of subjective pain ratings: after high-frequency and low-intensity TENS, the amplitude reduction of the N1, N2, and P2 waves was maximal when stimuli were delivered homotopically to the TENS; in low-frequency and high-intensity TENS, instead, their amplitude was similarly reduced regardless of which hand was stimulated (Fig. 2, Fig. 3, Fig. 4). Third, only low-frequency and high-intensity TENS resulted in long-lasting changes of ongoing brain activity, namely an enhancement of ongoing alpha oscillations in the primary sensorimotor cortex, maximally contralateral to the side of TENS application, and an increased functional connectivity between the primary sensorimotor cortex contralateral to the TENS and the mPFC (Fig. 5). This TENS-induced modulation of ongoing brain state might be the neurobiological basis for the more diffuse analgesic effect of low-frequency and high-intensity TENS.

Altogether, these results indicate that the two types of TENS act through different neurobiological mechanisms, which determine the different spatial features of the analgesic effect. These results can guide clinicians in choosing the appropriate set of TENS parameters to maximize the analgesic effect in different patients. Obviously, the application of these results in clinical routine will require testing their validity in different populations of patients with acute and chronic pain.

### 4.1 Neurobiological mechanisms of TENS induced analgesic effects

Evidence from animal studies demonstrates that high- and low-frequency TENS produce analgesic effects via different neurobiological mechanisms (DeSantana et al., 2010; Radhakrishnan et al., 2003). According to the gate control theory of pain, high-frequency and low-intensity TENS activates large-diameter Aβ fibers, which at dorsal horn level inhibit the incoming nociceptive volley transmitted via small-diameter, slow-conducting Aδ and C fibers innervating spatially-adjacent skin areas (Melzack and Wall, 1965). In line with this, we observed that high-frequency and low-intensity TENS produced a clear analgesic effect to homotopical nociceptive stimulation: the decrease of subjective ratings of pain intensity and unpleasantness, as well as laser-evoked brain responses, was significantly larger when nociceptive stimuli were delivered to the same hand where TENS was delivered (Fig. 2, Fig. 3, Fig. 4). Importantly, high-frequency and low-intensity TENS had a minimal analgesic effect even when nociceptive stimuli were delivered to the hand contralateral to the TENS side (pain intensity, on the 0–10 NRS: −1.82 ± 0.28 vs −0.22 ± 0.29, p = 0.0001; pain unpleasantness, on the 0–10 NRS: −1.57 ± 0.24 vs −0.61 ± 0.27, p < 0.001; Fig. 2). Thus, the analgesic effect of high-frequency and low-intensity TENS cannot be fully explained by a homotopical inhibition mechanism, and the concomitant contribution of a supraspinal descending inhibition mechanism remains a possibility, as suggested by several animal findings (Kalra et al., 2001; Sluka et al., 1999, 2005; Woolf et al., 1980). For example, it has been shown that the analgesic effect of high-frequency and low-intensity TENS can be reduced by spinalization (Woolf et al., 1980): although this high-frequency TENS delayed the response to the noxious stimulus in rats with complete spinal transection at the level of the 10th and 11th thoracic vertebrae, this antinociceptive effect was still present, but reduced compared to that observed in intact animals. In addition, the analgesic effect of high-frequency TENS was blocked by microinjection of the δ-opioid receptor antagonist naltrindole in both the spinal cord (Sluka et al., 1999, 2005) and RVM (Kalra et al., 2001).

In contrast to high-frequency and low-intensity TENS, which elicits non-painful tingling sensation, low-frequency and high-intensity TENS activates small-diameter Aδ and C nociceptive afferents, and therefore elicits tolerable but painful sensations. The tonic activation of Aδ and C afferents does not only elicit painful sensations, but also activates central nervous system structures resulting in analgesia (Bouhassira et al., 1987; Chung et al., 1984). Specifically, low-frequency and high-intensity TENS is thought to produce analgesia through the recruitment of descending pain inhibition system, via the activation of the PAG-RVM network (Kalra et al., 2001; Liebano et al., 2011; Zhao, 2008). Our observation that low-frequency and high-intensity TENS produced a spatially diffuse analgesic effect, not limited to the site of TENS application, agrees with this piece of knowledge. Indeed, subjective ratings of pain intensity and unpleasantness, as well as laser-evoked brain responses, were strongly reduced in the active TENS vs the sham condition when nociceptive stimuli were delivered both to the hand ipsilateral and contralateral to the TENS side (Fig. 2, Fig. 3, Fig. 4).

The differential effect of the two types of TENS on ongoing brain activity (Fig. 5) provides important mechanistic information. Only low-frequency and high-intensity TENS altered the ongoing brain state, and, specifically, significantly enhanced the amplitude of ongoing alpha oscillations at bilateral central electrodes (Fig. 5). That the amplitude of ongoing alpha oscillations influences the perceptual outcome of subsequently-delivered sensory stimuli has been consistently observed in several human studies (Kayser et al., 2016; Minami and Amano, 2017): the larger the alpha amplitude, the smaller the intensity of subjective perception and neural responses evoked by sensory stimuli (Tu et al., 2016). An important aspect is that the modulation of ongoing alpha oscillations was localized on bilateral central electrodes overlying the hand area of the primary sensorimotor cortex (Fig. 5). Given that alpha oscillations reflect the excitability of neuronal ensembles (Palva and Palva, 2007; Sadaghiani and Kleinschmidt, 2016), it follows that the analgesic effect of low-frequency TENS, consequent to the descending inhibition of nociception through the μ-opioid receptors in the PAG-RVM network (Zhang et al., 2014; Zhao, 2008), is likely to be triggered by the TENS-induced modulation of the functional state of the primary sensorimotor cortex, which could play an active role for the top-down inhibitory control of the nociceptive information (Ploner et al., 2017; Wutz et al., 2018). In support of this possibility, we observed an increase in functional connectivity between the primary sensorimotor cortex contralateral to the TENS and the mPFC, a core region of the descending pain inhibitory system, anatomically connected to the PAG (Fig. 5). This observation matches the increased activity in multiple cortical regions projecting to the PAG during the analgesia caused by DNIC, which is triggered by intense somatosensory stimuli similar to the low-frequency and high-intensity TENS used in the present study (Da Silva et al., 2018). In addition to the recruitment of the PAG-RVM network, low-frequency and high-intensity TENS could recruit the DNIC system, through the activation of neurons in the subnucleus reticularis dorsalis (SRD) in the caudal-dorsal medulla (Villanueva et al., 1996; Youssef et al., 2016b). Interestingly, the DNIC analgesic effect in humans is modulated by the strength of functional connectivity between SRD circuitry and prefrontal cortices (Youssef et al., 2016a). This observation provides an alternative explanation of the present results: the increased functional connectivity between the primary sensorimotor cortex contralateral to the TENS and the mPFC could lead to an enhanced descending inhibition through the connection between the mPFC and the SRD circuitry. However, given that the mPFC is involved in multiple functions, it is difficult to pinpoint a specific physiological mechanism for the observed effect.

### 4.2 Clinical implications

Once the results observed in this study, and particularly the spatially distinct effect of the two types of TENS, are replicated in clinical populations, their bedside application would be immediate. Indeed, most clinical studies of TENS have investigated the effect of different intensities and frequencies of stimulation while largely ignoring the possible influence of the body territory where TENS was applied, often assuming that the TENS electrodes should be placed in the proximity of the painful area (Baeumler et al., 2015). This assumption obviously limits the applicability of TENS in clinical practice, as in some conditions (e.g., patients with skin damage or visceral pain) placing the TENS electrodes close to the site of injury is problematic. The clear interaction between stimulation site and the type of TENS indicates that the maximal analgesic effect of high-frequency and low-intensity TENS is obtained only if the electrodes are placed near the painful area, whereas low-frequency and high-intensity TENS has an analgesic effect much less influenced by where the electrodes are located. Thus, our study provides important information to guide the selection of the best combination of stimulus parameters – intensity, frequency, and spatial location – to maximize the analgesic effect of TENS in clinical practice. Together with recent developments of neural markers for pain sensitivity across individuals (Hu and Iannetti, 2019), the current results make a step forward towards the implementation of personalized pain-relieving treatments.

In addition to provide an empirical basis for setting TENS parameters in future clinical studies, and potentially in clinical routine, our study helps explaining the conflicting data in previous animal and human reports. As already cogently highlighted (Mogil, 2009), inherent across-species differences make it difficult to explain the analgesic effect of TENS observed in humans using mechanisms inferred from animal models. Our observation of a significant analgesic effects consequent to the TENS-induced alteration of the state of the primary sensorimotor cortex is a mechanism that can be hardly recruited in animal studies, especially when using anaesthetized or “spinal” models (Garrison and Foreman, 1996).

## Author contributions

Conception and design of the study: WWP, ZYT, HL, YZK, and LH. Data acquisition and analysis: All authors. Drafting the manuscript: WWP, ZYT, GDI, and LH. All authors reviewed and accepted the final draft of the manuscript.

## Potential conflicts of interest

The authors have no conflict of interest to declare.
